# Gambling Disorder in the Digital Era: Clinical Challenges and Public Health Gaps in Brazil

**DOI:** 10.1192/j.eurpsy.2026.10799

**Published:** 2026-07-31

**Authors:** A. F. C. Ximenes, L. V. D. Araujo, P. F. B. de Araújo, A. B. D. C. Alves, L. C. Carneiro, M. I. O. de Morais, F. O. Barbosa, B. D. A. A. L. T. de Melo, E. D. F. Correia, V. L. de-Melo-Neto, D. B. B. Ferreira

**Affiliations:** 1 Psychiatry, Universidade Federal de Alagoas; 2 Psychiatry, Universidade de Ciências da Saúde de Alagoas, Maceió, Brazil

## Abstract

**Introduction:**

Gambling Disorder (GD) is the only behavioral addiction formally recognized by DSM-5-TR and ICD-11. Its prevalence has been increasing worldwide, driven by the expansion of online gambling, which enhances availability, anonymity, and exposure, leading to significant clinical, social, and economic consequences. High-income countries have already implemented surveillance and regulatory policies, but in Brazil data remain scarce and fragmented, hindering the design of prevention and care strategies. This gap justifies a comparative analysis between international and national evidence, aiming to inform both clinical practice and public health.

**Objectives:**

To review national and international evidence on GD, with emphasis on epidemiology, comorbidities, digital contexts, therapeutic strategies, and regulatory policies, highlighting implications for clinical practice and public health.

**Methods:**

A narrative review was conducted using PubMed and the Virtual Health Library (BVS). PubMed search (“Gambling disorder” AND “Online gambling”, 2020–2025) yielded 820 results; after screening, 17 were included. BVS search (“apostas” OR “transtorno do jogo” AND “transtorno mental” AND “Brasil”) retrieved 6 results, with 3 included for historical relevance. Inclusion: articles in English or Portuguese on epidemiology, comorbidities, digital environments, interventions, or policies. Exclusion: duplicates, case reports, or unrelated topics. Two reviewers screened and analyzed the studies. Findings were organized into five thematic axes: epidemiology, comorbidities, digital/social environments, interventions, and policies. Findings were thematically saturated across the selected studies.

**Results:**

International evidence shows that 46.2% of adults gambled in the past year, with 1.41% meeting criteria for problem gambling and 8.7% at risk. Online casinos and slot machines carry the highest association with problematic gambling. In Brazil, video poker players appear especially vulnerable, though national data remain scarce. Women progress more rapidly to GD (“telescoping effect”), particularly in rapid-luck games. GD strongly co-occurs with depression, anxiety, substance use, eating disorders, and compulsive behaviors. Cognitive-behavioral therapy shows moderate efficacy, while digital tools (feedback, self-exclusion, limit setting) are promising but heterogeneous. Regulatory frameworks in Switzerland and the UK emphasize harm reduction (e.g., reverse withdrawal bans, marketing limits, monitoring systems).

**Conclusions:**

GD is a multifaceted clinical and public health challenge. Priorities include national representative surveys, continuous harm surveillance, evidence-based digital therapies, regulation of gambling products and marketing, and multilevel policies addressing inequalities. In Brazil, the lack of representative data and specific regulations remains a critical barrier to prevention and care.

**Disclosure of Interest:**

None Declared

